# uPEPperoni: An online tool for upstream open reading frame location and analysis of transcript conservation

**DOI:** 10.1186/1471-2105-15-36

**Published:** 2014-02-01

**Authors:** Adam Skarshewski, Mitchell Stanton-Cook, Thomas Huber, Sumaya Al Mansoori, Ross Smith, Scott A Beatson, Joseph A Rothnagel

**Affiliations:** 1 School of Chemistry and Molecular Biosciences, University of Queensland, Brisbane, QLD 4072, Australia; 2 Research School of Chemistry, Australian National University, Canberra, ACT 0200, Australia

**Keywords:** 5′UTR, uORFs, mRNAs, Sequence conservation, Short peptides, Poly-cistronic, Homology heatmaps

## Abstract

**Background:**

Several small open reading frames located within the 5′ untranslated regions of mRNAs have recently been shown to be translated. In humans, about 50% of mRNAs contain at least one upstream open reading frame representing a large resource of coding potential. We propose that some upstream open reading frames encode peptides that are functional and contribute to proteome complexity in humans and other organisms. We use the term uPEPs to describe peptides encoded by upstream open reading frames.

**Results:**

We have developed an online tool, termed uPEPperoni, to facilitate the identification of putative bioactive peptides. uPEPperoni detects conserved upstream open reading frames in eukaryotic transcripts by comparing query nucleotide sequences against mRNA sequences within the NCBI RefSeq database. The algorithm first locates the main coding sequence and then searches for open reading frames 5′ to the main start codon which are subsequently analysed for conservation. uPEPperoni also determines the substitution frequency for both the upstream open reading frames and the main coding sequence. In addition, the uPEPperoni tool produces sequence identity heatmaps which allow rapid visual inspection of conserved regions in paired mRNAs.

**Conclusions:**

uPEPperoni features user-nominated settings including, nucleotide match/mismatch, gap penalties, Ka/Ks ratios and output mode. The heatmap output shows levels of identity between any two sequences and provides easy recognition of conserved regions. Furthermore, this web tool allows comparison of evolutionary pressures acting on the upstream open reading frame against other regions of the mRNA. Additionally, the heatmap web applet can also be used to visualise the degree of conservation in any pair of sequences. uPEPperoni is freely available on an interactive web server at http://upep-scmb.biosci.uq.edu.au.

## Background

The discovery of mutations in upstream Open Reading Frames (uORFs) associated with disease [1] has brought renewed interest in uORFs and the peptides they encode. Bioinformatic analyses of cDNA and EST databases have estimated that up to 50% of all eukaryote mRNAs contain upstream AUG (uAUG)/uORFs within the 5′ untranslated region (5′UTR) [2-8]. Recent ribosome profiling studies have indicated that many of these uAUGs are recognised by scanning ribosomes suggesting that their associated uORFs are translated [9-11]. To date, 29 peptides encoded by uORFs have been identified in proteomic studies [12-14] although there is currently no information on their functions. We have previously proposed that part of the eukaryotic proteome is composed of peptides resulting from the translation of uORFs [2].

The canonical role for uAUGs/uORFs is the regulation of protein expression by modulating translation of the main open reading frame (mORF), which is usually the longest coding sequence (CDS) present on a mRNA. In most cases uAUGs/uORFs lower translation of the mORF by reducing the number of ribosomes reaching and initiating at the main AUG start codon [1,15-18]. While there are many reports of uORFs reducing translation of the CDS [1,16,18], only a few studies have investigated the potential of uORFs to generate bioactive peptides [2,12,19,20]. We use the term uPEPs to describe their origin as uORF-derived peptides.

Searches for cross-species conservation of uORFs can reveal those that encode potential functionally important peptides [2,12,19,20]. High levels of sequence identity between uORF homologues (when compared to the mRNA as a whole) are an indication that the encoded uPEP has been maintained during evolution. Furthermore, protein coding regions generally have more synonymous substitutions than non-synonymous mutations, and that this observation can be used to predict potential protein coding regions [21]. The algorithms presented here screen uORFs for these characteristics in order to identify those encoding potential uPEPs [2]. The uPEPperoni program also includes an algorithm that produces sequence identity heatmaps which allow rapid visual inspection of conserved regions in paired mRNAs.

## Implementation

**Figure 1:**
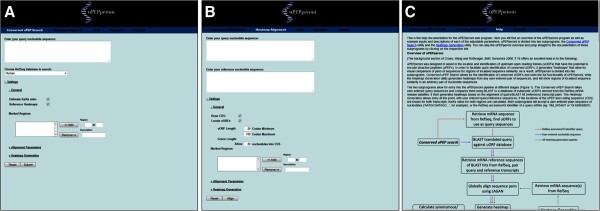
**Screenshots of the search, alignment and help pages of uPEPperoni. (A)** The conserved uPEP search page showing the user-selectable settings for the RefSeq database, Ka/Ks ratio, reference heatmaps, alignment parameters and heatmap generation. **(B)** The heatmap alignment page showing the user-selectable settings for visual representation of the main coding sequence (CDS) and uORFs and the search parameters for uORF-length, the extent of uORF overlap into the CDS and the region of the transcript to be searched. **(C)** The help page. uPEPperoni is hosted on an Apache server on a Linux platform and is publically accessible free of charge at http://upep-scmb.biosci.uq.edu.au. Full documentation of uPEPperoni is also accessible via links on the website. The uORF reference database is automatically rebuilt on the server shortly after each major RefSeq release. We archive previous uORF reference databases. The RefSeq release version number from which the reference database is derived is shown on the web page.

The uPEPperoni web application is divided into three separate utilities; a *conserved uPEP search* utility, a *heatmap generation* utility and an *update* utility (Figure 1). The *conserved uPEP search* utility takes a given query sequence or RefSeq accession number, locates uORFs based on given parameters such as uORF length and allowed distance into the mORF, then compares these uORFs against a selected reference uORF database. The uORF database is derived from the eukaryote mRNA datafiles of NCBI’s RefSeq Database major release. uPEPperoni will automatically update its uORF databases to reflect new RefSeq releases. RefSeq sequences where the start of the mORF is not defined are excluded during the uORF database building step.

## Results and discussion

To identify conserved uPEPs, a query sequence is aligned against reference uORFs using the tblastx subprogram of NCBI’s blastall standalone executable. The tblastx subprogram is used in preference to nucleotide based blast programs because of its better sensitivity and to preference selection of uPEPs conserved at the amino acid level, rather than uORFs conserved at the nucleotide level. Individual transcripts from the uORF database that are found to contain a putative uPEP homologue are paired with the query sequence, and the pair passed to the *heatmap generation* utility. As an alternative to receiving input sequences from the *conserved uPEP search* utility, the *heatmap generation* utility can accept user entered query/reference nucleotide sequences directly.

The mRNA sequences for each conserved query/reference uORF pair are aligned pairwise using the LAGAN toolkit [22], with match/mismatch scores and gap penalties specified by the user. We normally use a gap opening penalty of 50, no gap extension penalty, +5 for a nucleotide match and -4 for a mismatch as default parameters. Given a query sequence (*Q*) of length *q*, and a reference sequence (*R*)*,* the alignment produces three sequences of equal length (*m*). These are; the aligned query (*Q’*) and aligned reference sequences (*R’*), comprising the query and reference sequences with alignment gaps inserted, and a match sequence (M) derived by assigning 1 to the *i*^*th*^ element, if the *i*^*th*^ element of the *Q’* and *R’* are a nucleotide match, and assigning 0 if otherwise.

The percentage identity of a region surrounding a nucleotide in *Q* can be calculated from *M* and *Q’*. If *z* is the integer part of *w/2*, where *w* is the size of the window which specifies the region of calculation when centred on a nucleotide in *Q’*, then for each non-gap element *Q’*_*i*_ in *Q’*, a percentage identity is calculated by the following:

$$f\left(Q_{i}^{\prime }\right)=\left\{\begin{array}{cc} \frac{1}{z+m-i+1}\sum_{j=-z}^{m-i}M_{i+j}, & i+z>m\\ \frac{1}{z+i}\sum_{j=1-i}^{z}M_{i+j}, & i-z\leq 0\\ \frac{1}{2z+1}\sum_{j=-z}^{z}M_{i+j}, & \mathrm{otherwise} \end{array}\right)$$

**Figure 2:**
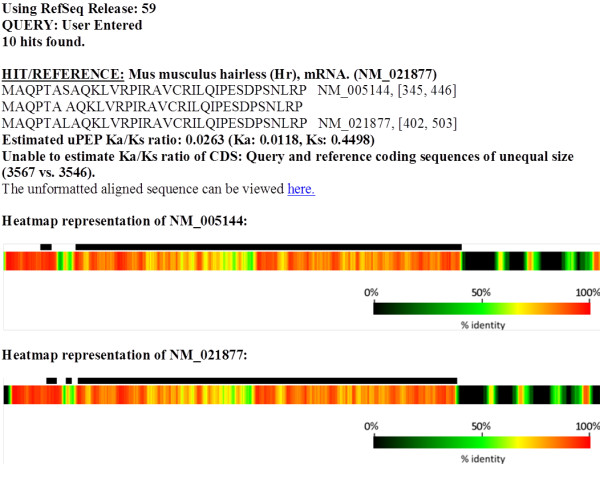
**Example output showing the heatmaps produced by querying the mRNA sequence of the *Homo sapiens *Hairless (*HR*) transcript (NM_005144) against *Mus musculus *Hairless (*Hr*) (NM_021877).** The solid bars above the heatmap indicate the ORFs on the transcript. The output lists the most conserved uPEPs first. The heatmap generated by the query sequence is shown first; in this case human *HR* aligned with mouse *Hr* transcript. The reciprocal heatmap generated using the reference sequence is shown below (mouse *Hr* transcript versus human *HR*). The inclusion of the Reference Alignment is selectable by the user. The unformatted aligned sequence can be viewed using a hyperlink shown above the heatmap.

Placing the value of *f* (*Q’*_*i*_) into a vector (*P*) for each non-gap element *Q’*_*i*_ results in a vector of length *q*. Every element of P is then correlated to a reference heat gradient, which produces a heatmap. Heatmaps are used to visualise both the extent and degree of sequence identity between *Q* and *R*, and allow comparison between different regions, such as the 5′ and 3′ UTRs, uORFs and the mORF in any pair of transcripts. Moreover, they also allow rapid inspection for other conserved *cis*-elements such as miRNA target sequences and splicing regulatory elements. An example heatmap is shown in Figure 2.

In addition, uPEPperoni estimates the ratio of synonymous to non-synonymous substitution rates of the mORF and uORF using the method of Yang and Nielsen [23], implemented in a library compiled from modified source code of the yn00 program in the PAML package [24]. As synonymous substitutions are favoured in protein coding sequences, the ratio provides additional confidence on the likelihood of any given uORF to encode a bioactive peptide. Furthermore, the synonymous to non-synonymous substitution ratio of the mORF provides an internal control to which the uORF ratio can be compared, allowing for an evaluation of selective pressures on both the uORF and mORF.

**Figure 3:**
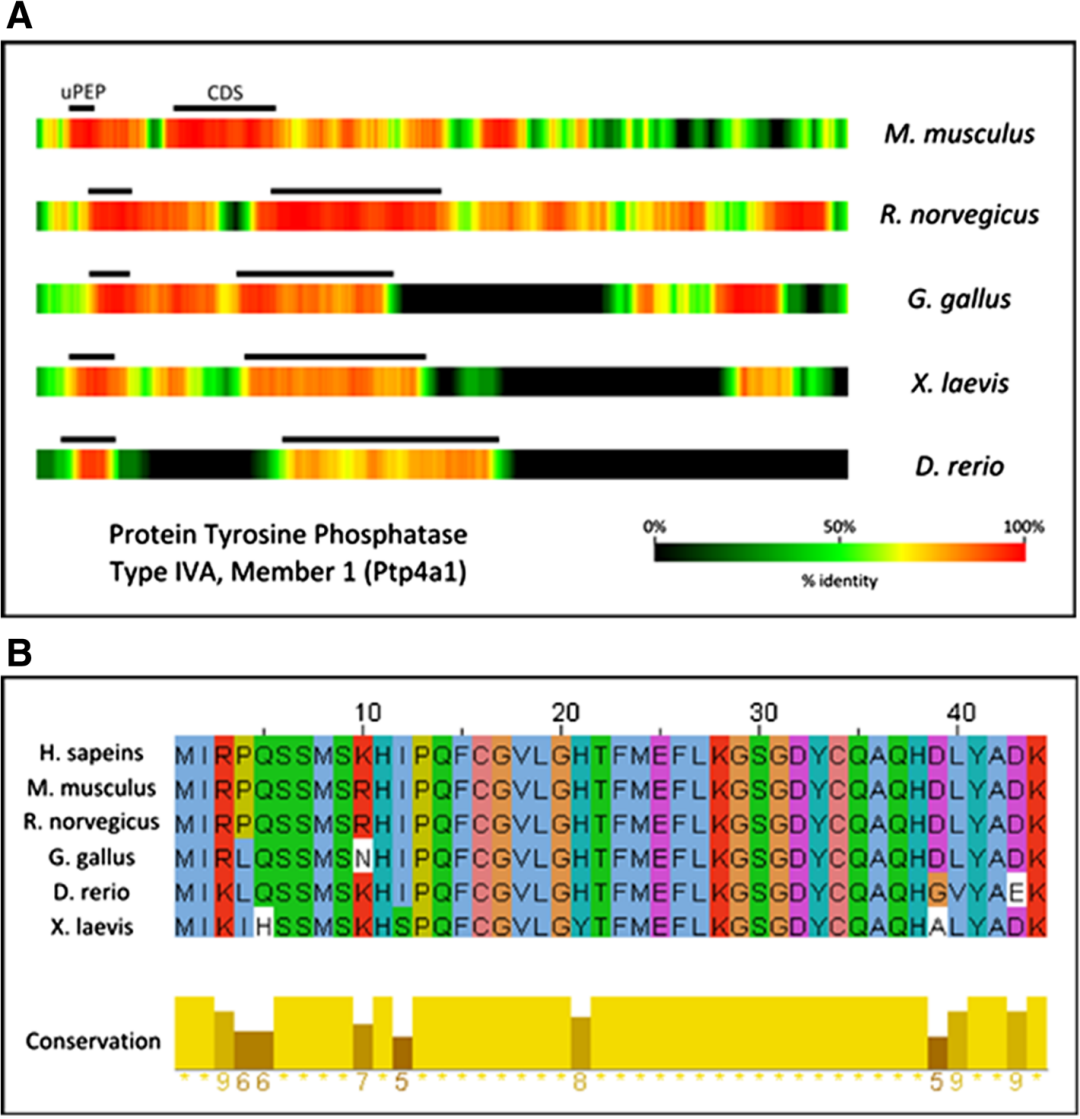
**Several heatmaps of aligned transcript-pairs can be combined to provide a visual snapshot of sequence conservation. (A)** Heatmaps for each pair-wise analysis of the human transcript encoding protein tyrosine phosphatase type IVA, member 1 (Ptp4a1) (NM_003463) with the othologous non-human transcript are shown. Black lines above each heatmap mark the position of the conserved uPEP and CDS for that species. Note the conservation of this uPEP even as the phylogenetic distance between the comparison species (on the right) widens. **(B)** ClustalW alignment of the *Ptp4a1* uPEP, translated *in silico* from the conserved uORF. The numbers below the bar graph represent the conservation of each individual amino acid, where 10 (shown as an asterisk (*)) indicates identity across all species.

In order to test the program, we employed uPEPperoni to re-examine the conserved uORFs found in our original study [2]. We confirmed the conservation of 202 uORFs and identified an additional 19 others. The conserved uORFs were confirmed using ORFfinder [25]. Importantly, with uPEPperoni we were able to extend the number of species in which conservation was found to 13 (Table 1); 147 showed conservation across several species while 55 uPEPs showed conservation between human and mouse only. The utility of this program is underscored by the output shown in Figure 3A. Here we examined the third uORF present on the *Ptp4a1* transcript using uPEPperoni to make pair-wise comparisons between the human transcript and orthologs in mouse, rat, chicken, frog and fish. The heatmap compilation allows a quick visual assessment on the degree of sequence identity and readily shows the conservation of uPEP sequence. The high level of conservation in multiple species identified by uPEPperoni was confirmed using ClustalW and shows the maintenance of this peptide over relatively large evolutionary distances (Figure 3B).

**Table 1:** **List of species with one or more conserved uPEPs using the uORFs identified in Crowe *et al*** [2]

**Species containing one or more conserved uPEPs**	**Number of conserved uPEPs**^**a**^
Human, mouse, rat, cow, chicken, frog, monkey, horse, chimpanzee, zebra fish, salmon	1
Human, mouse, rat, orangutan, chicken, frog, zebra fish, salmon	1
Human, mouse, rat, cow, monkey, chicken, rabbit, chimpanzee	1
Human, mouse, rat, pig, chicken, cat, horse	1
Human, mouse, rat, cow, orangutan, monkey	1
Human, mouse, rat, cow, orangutan, pig	1
Human, mouse, rat, cow, orangutan, frog	1
Human, mouse, rat, cow, chicken, frog	1
Human, mouse, rat, cow, orangutan	13
Human, mouse, rat, orangutan, chicken	1
Human, mouse, rat, zebra fish, frog	1
Human, mouse, rat, orangutan, pig	1
Human, mouse, rat, pig, monkey	1
Human, mouse, cow, pig, orangutan	1
Human, mouse, rat, orangutan	10
Human, mouse, rat, cow, monkey	2
Human, mouse, rat, cow, pig	1
Human, mouse, rat, cow, chicken	1
Human, mouse, rat, cow, frog	1
Human, mouse, rat, cow	27
Human, mouse, cow, orangutan	7
Human, mouse, cow, pig	3
Human, mouse, rat, pig	2
Human, mouse, rat, monkey	2
Human, mouse, cow, monkey	1
Human, mouse, orangutan, chimpanzee	1
Human, mouse, orangutan, hamster	1
Human, mouse, rat, horse	1
Human, mouse, rat, chicken	1
Human, mouse, rat	36
Human, mouse, cow	15
Human, mouse, orangutan	5
Human, mouse, pig	2
Human, mouse, monkey	1
Human, mouse	55

^a^Specifies the total number of individual uPEPs that show sequence conservation across the group of species indicated.

## Conclusions

We have developed a web tool that facilitates the identification of conserved uORFs. This tool alleviates the need to use several single-facet programs for the detection of uPEPs. UPEPperoni can be used to populate the databases employed in the identification of novel small peptides by mass spectrometry and enhance the discovery of a novel source of regulatory molecules. Given the renewed interest in the role of uORFs in human disease [1] and the possibility that peptides encoded by uORFs can have functionality beyond regulation of translation [2,13,26], uPEPperoni offers improved utility in their identification and will aid in their characterisation.

## Availability and requirements

• **Project name:** uPEPperoni: An online tool for upstream open reading frame location and analysis of transcript conservation.

• **Project home page:**http://upep-scmb.biosci.uq.edu.au.

• **Operating system(s):** Platform independent.

• **Programming language:** Server-side: Perl, C, Python, C++, HTML and JavaScript.

• **Other requirements:** None.

• **License:** Not applicable.

• **Any restrictions to use by non-academics:** None.

## Abbreviations

uAUG: upstream start codon; uORF: upstream open reading frame; mORF: main open reading frame; uPEP: uORF-encoded peptide; 5′ UTR: Five prime untranslated region; 3′ UTR: Three prime untranslated region; CDS: Coding DNA Sequence (synonymous with mORF).

## Competing interests

The authors declare that they have no competing interests.

## Authors’ contributions

AS wrote the code and acquired data. MS-C revised and updated the code. SAM tested the tool and acquired data. TH, SB and JR participated in the design of the study and helped draft the manuscript. JR conceived the study. All authors have read and approved the final manuscript.
